# 

**Published:** 1837-10

**Authors:** 


### BOOKS RECEIVED FOR REVIEW.

ENGLISH.

1. The American Medical Library and Intelligencer; a concentrated Record of Medical Science and Literature. Edited by Robley Dunglison, M.D., Professor of the Institutes of Medicine, &c. in Jefferson College, Philadelphia, &c. Vol. I. No. I.—VIII. Published Semi-monthly. —Philadelphia, 1837. 8vo. Price ten dollars a year.

2. The Medical Student; or, Aids to the Study of Medicine: including a Glossary of the Terms of Science, and of the Mode of Prescribing; Bibliographical Notices of Medical Works, &c. By R. Dunglison, M.D., Professor, &c.—Philadelphia, 1837. 8vo. pp. 323.

3. An Examination of Phrenology; in two Lectures delivered to the Students of Columbia College. By Th. Sewall, M.D., Professor of Anatomy and Physiology.—Washington, 1837. 8vo. pp. 67; with 8 Plates.

4. Reports on the Botany, Zoology, Mineralogy, and Geology of the State of New York, from official Surveys. Published by Government.—Albany, 1837. 8vo. pp. 212.

5. Address to the Medical Graduates of the Jefferson Medical College. Delivered March 11, 1837, by R. Dunglison, M.D., Professor of the Institutes of Medicine, &c. —Philadelphia, 1837. 8vo. pp. 20.

6. Oration on the Guidance of a sound Philosophical Spirit in the Investigation of Medical Science. Read before the Cincinnati Medical Society, January, 1837, by J. P. Harrison, M.D. Professor of Materia Med.—Cincinnati, 1837. 8vo. pp. 22.

7. Plates of the Cerebro-Spinal Nerves, with References; for the use of Medical Students. By P. B. Goddard, M.D., Prosector of Anatomy in the University of Pennsylvania.—Philadelphia, 1837. 4to. pp. 60; 12 Plates.

8. A Translation of the Pharmacopœia Londinensis of 1836, with Descriptive and Explanatory Notes of the Materia Medica, &c. By Th. Castle, M.D. F.L.S.—London, 1837. 18mo. pp. 260. 5s.

9. Observations on the Topography, Climate, and prevalent Diseases of the Island of Jersey; the Result of meteorological Observations and general Practice during thirteen Years. By G. S. Hooper, M.D.—London, 1837. 8vo. pp. 199. 6s.

10. A Treatise on the Influenza of 1837; containing an Analysis of 100 Cases observed at Birmingham, between 1st January and 15th January. By Peyton Blakiston, M.A. M.L. Cant., Physician to the Birmingham Dispensary, &c.—London, 1837. 8vo. pp. 60.

11. A Treatise on the Malformations, Injuries, and Diseases of the Rectum and Anus. Illustrated by Plates. By George Bushe, M.D.—New York, 1837. 8vo. pp. 299.

12. The Philosophy of the Eye; being a familiar Exposition of its Mechanism and of the Phenomena of Vision, with a View to the Evidence of Design. By John Walker, Lecturer on the Eye in the Manchester School of Medicine, &c. With numerous Illustrations.—London, 1837. 8vo. pp. 300.

13. Medical Relief for the Labouring Classes, on the Principle of Mutual Assurance. By H. W. Rumsey, one of the Surgeons of the Chesham Self-supporting Dispensary.—London, 1837. 12mo. pp. 69. 1s. 6d.

14. On a New Mode of Treatment employed in the Cure of various Forms of Ulcer and granulating Wounds. By F. C. Skey, F.R.S., Assistant Surgeon to Saint

Bartholomew's Hospital, &c. — London, 1837. 8vo. pp. 85.

15. The Physician's Vade Mecum; or, Manual of the Principles and Practice of Physic. By Robert Hooper, M.D. New Edition, considerably enlarged and improved, by Michael Ryan, M.D.—London, 1837. 8vo. pp. 417. 7s. 6d.

16. On the Nature and Treatment of Diseases of the Heart; with some New Views on the Physiology of the Circulation. By James Wardrop, M.D. &c.—London, 1837. 8vo. pp. vi. 100, 20. Part I. Four Plates.

17. Memoirs on the Nerves. By Marshall Hall, M.D. F.R.S. L. & E.—London, 1837. 4to. With Plates.

18. Surgical Observations on Tumours, with Cases and Observations. By J. C. Warren, M.D., Professor of Anatomy and Surgery in Harvard University.—Boston, 1837. 8vo. pp. 607; with 16 Plates.

19. Text-Book of a Course of Lectures on the Theory and Practice of Physic; for the use of the Medical Students of Harvard University. By James Jackson, M.D.—Boston. Part I. 1825. Part II. 1827.—8vo. pp. 96, 227.

20. An Experimental Essay on the relative Physiological and Medicinal Properties of Iodine and its Compounds; being the Harveian Prize Dissertation for 1837. By Charles Cogswell, A.B. M.D. &c. —Edinburgh, 1837. 8vo. pp. 179. 5s.

21. Notice of Patents granted to Joseph Amesbury, Surgeon, for certain Apparatus used in the Treatment of the Deformity of the Spine, &c.—London, 1837. 8vo. pp. 24. 1s. 6d.

22. Eloge upon Baron G. Dupuytren. By E. Pariset. Translated from the French, with Notes, by J. I. Ikin, Surgeon.—London, 1837. 8vo. pp. 60. 2s. 6d.

23. Inaugural Dissertation on the Physiology and Pathology of the Brain; being an Attempt to ascertain what Portions of that Organ are more immediately connected with Motion, Sensation, and Intelligence. Submitted to the Medical Faculty of the University of Edinburgh. By J. H. Bennett.—Edinburgh, 1837. 8vo. pp. 79. 2s. 6d.

24. First Principles of Medicine. By Archibald Billing, M.D. A.M. &c. Second Edition.—London, 1837. 8vo. pp. 130.

25. Advice on the Care of the Teeth. By E. Saunders, Dentist. — London, 1837. 12mo. pp. 107.

26. An Essay on Pyrexia, or Symptomatic Fever, as illustrative of the Nature of Fever in general. By H. Clutterbuck, M.D.—8vo. pp. 136.

27. A Medico-legal Treatise on Homicide by external Violence: with an Account of the Circumstances which modify the Medico-legal Characters of Injuries and exculpatory Pleas. By Alexander Watson, Esq., Fellow of the R. Coll. of Surgeons, Edinb.—Edinburgh, 1837. 8vo. pp. 355. 9s.

28. The Theory of Electric Repulsion examined, in a Series of Experiments on certain Properties attributable to the Elements which constitute Electric Excitation, adduced principally to show the Non-Existence of Repulsion. By Charles Hales. —London, 1837. 8vo. pp. 22.

29. The Nature and Treatment of Diseases of the Ear. By Dr. Wm. Kramer. Translated from the German, with the latest Improvements of the Author since the last German Edition. By J. R. Bennett, M.D., Physician to the General Dispensary, Aldersgate street. — London, 1837. 8vo. pp. 306.

FOREIGN.

1. Die Pest des Orients, wie sie entsteht und verhütet wird; drei bucher von Dr. C. J. Lorinser, &c.—Berlin, 1837. 8vo. pp. 461.

2. Boeken-Verslag tot aanwijzing van den inhoud en de strekking van nieuw uitkomende Werken; zonder recensie. Voor, 1836.—Breda. 8vo. pp. 256.

3. Genees - en heelkundige bijdragen. Verzameld door C. J. Nieuwenhuijs, M.D. &c. Eerste stuk.—Amsterdam, 1837.—8vo. pp. 113.

4. Verhandeling over de Percussie en Auscultatie, door E. C. Büchner, &c.—Amsterdam, 1837. 8vo. pp. 143.

5. Marienbad, seine Heilquellen und Umgebungen. Von J. A. Frankl, M.D. &c. —Prag. 1837. 8vo. 175.

6. Oratio quam in Conventu Societatis Physico-medicæ Mosquensis D. xiv. Aprilis, 1836, anni habuit D. M. Marcus Societatis præses.

7. Die neuesten Entdeckungen in der Materia Medica. Für praktische Aerzte geordnet, von Dr. Joh. Heinrich Dierbach, Professor der Medicin zu Heidelberg.—Heidelberg, 1837. 8vo. pp. 656.

8. Il Filiatre-Sebezio. Diretto dal Commendator Ronchi. Redatto dal prof. Salvatore de Renzi. Vol. xii. xiii.—Napoli, 1835-6.

9. Cenni sul Cholera-Morbus e sul metodo di cura. Dal Dottore Stanislao Orazi. —Loreto, 1836. 8vo. pp. 66.

10. Reflessioni sulla corrente malattia Asiatico-Cholerica e modo sicuro di preservarsene del Dottore Palmazio Lengi.—Livorno, 1835. 8vo. pp. 40.

11. Cenni sul Cholera-Morbus Asiatico del Dottore Christofano Rasis.—Livorno, 1835. 8vo. pp. 31.

12. Il Cholera a Roda Racconto istruttivo.—Firenze, 1835. 8vo. pp. 28.

6

### INDEX TO VOL. IV.

OF THE

### BRITISH AND FOREIGN MEDICAL REVIEW.

---

PAGE

ABSORBENT system of vegetables . 15
Acarus of the itch, on the . . 513
Addison, Dr. on pneumonia . 356
Albers, Dr. on inhalation of chlorine 212
Ammon, Dr. on ophthalmology . 480
Amputation of the limbs in utero . 467
Anatomy, morbid, Dr. Hodgkin on . 1
general, manual of . 497
Andral on diseases of the chest . 285
Aneurism, varicose, M. Perry on . 138
popliteal, case of . 256
Aorta, on pulsations of . . 533
Apothecaries in Norway . . 546
Ashwell, Dr. his obstetric cases . 369
Arachnoid membrane, morbid anatomy of . . . . . 40
Areola as a sign of pregnancy . 452
Aretæus on medicine, translation of 490
Arsenic, on iron as the antidote of . 237
Arteries, Dr. Beck, on the ligature of 154
Association, British . . 567
Atlas, fracture and displacement of . 141
Auscultation in pregnancy . 457

Bath, on the nitro-muriatic acid . 254
Baths, Turkish, account of . . 388
Beck, Dr. on infanticide . . 87
Beck, Dr. K. on ligature of arteries 154
Bigger, Dr. on transplanting the cornea . . . . . 537
Bone, atrophy of, Mr. Curling on . 152
Bones, on the reparation of . 367
BOOKS for Review . . 283, 569
Botany, various works on . . 1
Bow, Dr. on opium in croup . 243
Black expectoration, Dr. Thomson on 150
Blisters, Dr. Collier on . . 113
Brain of the negro, on the size of . 529
Mr. Solly's anatomy of . 485
Dr. Nasse on diseases of . 427
on tumours in . . 376
Breschet on the lymphatic system . 325
Brigham, Dr. on religion and education . . . . . 55
Bright, Dr. on cerebral tumours . 376
Brodie, Sir B. on the spinal cord . 143
on nervous diseases . 132

PAGE

Bronchi, obstruction of . . 300
Bronchi, dilatation of . . 309
Bronchitis, Dr. Stokes on . . 398
Bruit de diable, Dr. Ward on . 245
Brown, Mr. on glanders in man . 255
Buffy coat, during pregnancy . 459
Burne, Dr. on inflamed cæcum . 148
Bushe, Dr. on diseases of the rectum 467

Calculus, Dr. Hodgkin on . 375
Cataract, on the extraction of . 257
Carpenter, Mr. on instinctive actions 530
on unity of function 532
Cæcum, on inflammation of . 148
Cæsarean section, four times performed, 521
Cerebral murmur, Dr. Smyth on . 536
Chemistry, Mr. Ede's Facts in . 498
Chest, Dr. Stokes on diseases of the 285
China, medical missions to . 568
Chlorine gas, inhalation of . 212, 224
Cholera, Aretæus on . . 491
Christiania, university of . . 541
Churchill, Dr. on the umbilical cord 261
Clubfoot, on the treatment of . 231, 539
Circulation, influence of gravity on . 246
Colchicum, Dr. Lewins on . 249, 565
its external use in gout 536
Collier's, Dr. translation of the Pharmacopœia . . . 101
Colquhoun, Mr. on animal magnetism 441
Comparison, its value in auscultation 296
Convulsions, puerperal . 406, 408
Cooper, Mr. B. on the reparation of bone . . . . 367
Cord, umbilical, on the length of . 261
Cornea, on the transplantation of . 557
Corpus luteum, state of, in pregnancy 461
Craigie, Dr. on fever in Edinburgh . 247
Creosote, its use in various diseases 251
Cummin, Dr. on infanticide . 87
Curling, Mr. on atrophy of bone . 152
Cyclopædia of Surgery, Mr. Costello's, 195

Death, on the signs of . . 197
Decandolle, M. his Botany . 1
Delivery, on the signs of . . 465
Diagnosis, on the uncertainty of 478

PAGE
Diabetes cured by diuretics . 254
Diaphragm, laceration of . . 260
Dictionary of Medicine, Dr. Copland's 200
Digestion, artificial, experiments on 201
Diseases of the chest, Dr. Stokes on 285
physical signs of 290
Dissection wounds, treatment of . 140
Dunglison, Dr. his American Library 495
Medical Student 496
Dyspepsia, on nux vomica in . 244

Ede, Mr. his Facts in Chemistry . 498
Edinburgh, graduations at . 563
Education, influence of, on health . 55
Emetic tartar in inflamed mamma . 533
Emphysema of the lungs . . 311
Empyema, Dr. Hodgkin on . . 44
Epistaxis, new mode of stopping . 231
Erysipelas, on the tonic treatment of 245
Evans, Mr. on West India fevers . 160
Excretions of plants . . 27
Eye, on the action of the muscles of 477
its importance . . 480
Mr. Walker's Philosophy of . 496
Eyelid, lower, on the formation of . 483

Fever, spotted, Dr. Wilson on . 535
at Limerick, Dr. Geary on . 536
puerperal, treated with ice . 517
complicated with bronchitis . 303
in the Edinburgh Infirmary . 247
endemic, West Indian . 160
Fingers, contraction of, new variety of, 171
on a spasmodic affection of 507
Fischer, his treatment of diseases of the eye . . . . 481
Fracture of the skull during delivery . 96
Fricke, Dr. on varicocele . . 232
Froriep, Dr. his Surgical Copper-plates, 168
Function, on the unity of . . 532

Gangrene of serous membranes . 52
Geddes, Mr. on hepatic abscess . 279
Gestation, human, period of . 463
Glanders in man, case of . . 255
Glands, lymphatic, history of . 333
physiology of . 340
pathology of . 343
Goddard, Dr. his plates of the nerves 497
Gonorrhœa in women, mode of treating . . . 258, 540
Gout, castor-oil frictions in . 243
Graduations at Edinburgh . . 563
Gravity, influence of, on the circulation . . . . . 246
Green, Dr. on diseases of the skin . 175
Griffin, Dr. on spinal irritation . 252
Gout, castor-oil frictions in . 243
Gully, Dr. on neuropathy . 471
Guy's Hospital Reports . . 349

Hannay, Dr. on gonorrhœa . . 258

Harem, medical visit to . . 385

PAGE
Harrison, Dr. on tubercles . . 244
Hastings, Dr. on natural history . 197
Hamilton, Dr. his observations on midwifery . . 181, 398
Headach, on the cure of . . 251
Heart, valves of, their functions . 364
Heidelberg, a letter from . . 282
Heim, Dr. his medical works . 492
Hemorrhage, uterine, Dr. Hamilton on 406
Henslow, Mr. his Botany . 1
Hepatic abscess in India . . 279
Hernia, on the operation for . 256
diaphragmatic, case of . 479
pericardii, case of . 532
Hodgkin, Dr. on calculus . . 375
his Morbid Anatomy . 31
Hoffman, Mr. on Medical Jurisprudence . . . . 489
Holst, Dr. on Medicine in Norway 541
Hooper, Dr. on the climate of Jersey 494
Hospitals in Norway . . 547
Hunter, John, his Works and Life . 75
account of his death . 84
William, account of . 76, 189
on Infanticide . 87
Hunterian oration, Sir B. Brodie's . 189
Hydrocephalus, Mr. Mayo on . 246
Hydrocyanic acid, poisoning by . 533
Hydrophobia, researches on . 240
Hydrostatic test, value of . . 90
Hysterical affections, on local . 135

Ileus, treatment of, by belladonna . 220
Impregnation of the mammifera, Mr. Jones on . . . . 527
plants, on the 29, 561
Infanticide, Cummin and Schwörer on 87
cases of . . 521
Ingleby, his cases in Midwifery . 398
Inoculation of morphia . . 506
Insects, Mr. Newport on the temperature of . . . . 527
Instinctive actions, Mr. Carpenter on 530
Intestines, on the suture of . 512
Iridoncosis, Dr. Klemmer on . 482
Iris, on staphyloma of . . 482
Iron, oxyde of, an antidote for arsenic, 237
Isis Revelata, or animal magnetism 441
Itch, nature and treatment of, 513, 515

Jahn, Dr. his new system of pathology 120
Jersey, Dr. Hooper on the climate of 494
Joints, on the excision of . . 230
Jones, Mr. W. on impregnation . 527

Key, Mr. on lithotrity and lithotomy 350
King, Mr. on the valves of the heart 364
Knee-joint, contraction of the . 169
Knox, Dr. on the pulse . . 241

Labours, laborious . . . 400
Laennec, Dr. Mer. on auscultation . 285
Larynx and trachæa, works on the . ib.

PAGE
Lead, acetate, on the action of . 208
Lectures, American . . 194
Leeches, their application in headach 251
Lefevre, Dr. on Medicine in Russia 263
Lendrick, Dr. on the nitro-muriatic bath . . . . 254
Lepra in Russia . . . 227
Lewins, Dr. on colchicum . 249, 565
Library, the American Medical . 495
Lindley's Botany . . . 1
Liston, Mr. on tumours of the mouth 147
Lithotomy, healing of the wound in . 539
and lithotrity, Mr. Key on 350
Lunatic asylums in Turkey . 390
Lungs, Dr. Stokes on diseases of . 285
case of rupture of . . 254
Luxation of the humerus, case of . 256
Lymph, motion of its globules . 500
Lymphatics, M. Breschet on the . 325
literary history of . 326
comparative anatomy of 338

Madness, acute, rare in Turkey . 390
Magnetism, animal, Mr. Colquhoun on 441
Malcolmson, Mr. on solitary confinement . . . . 191
Mamma, inflamed, treatment of . 533
hypertrophy of . 224
Mayo, Dr. on hydrocephalus . 246
Meckel, M. his Manual of Anatomy 497
Medical jurisprudence, Mr. Hoffman on 489
Medulla oblongata and spinal cord . 486
Membranes, serous, Dr. Hodgkin on 31
false, Dr. Hodgkin on . 36
Memory, curious case of loss of . 478
Menses, suppression of, a sign of pregnancy . . . . 451
Midwifery, Dr. Hamilton and Mr. Ingleby on . . 181, 398
Dr. Ashwell's cases in 369
Midwives in Norway . . 547
Mitscherlich, Prof. on acetate of lead 208
Mollison, Dr. on the pulmonic pulse 241
Montgomery, Dr. on the signs of pregnancy . . . 448
Morgan, Mr. his Surgery . . 185
Morphine, on the inoculation of . 506
Mortification of the toes . . 259
Moxa, on the use of . . 217
Müller, Prof. on artificial digestion 201
Muscles, state of, in spinal curvature 509

Nasse, Drs. their physiological researches . . . . 414
Negro, size of his brain . . 529
population, statistics of . 261
Nerves, plates of the cerebro-spinal 497
fifth pair, origin of . 212
Nervous affections, Sir B. Brodie on 132
Neuropathy, or Nervousness, Dr. Gully on . . . . 471
Newport, Mr. on the temperature of insects . . . . 527

PAGE
Nipple and areola in pregnancy . 452
Norway, state of medicine in . 541
Nutriment of vegetables . . 16
Nux vomica in dyspepsia . . 244

Old persons, pneumonia of . 501
Ophthalmology, articles in . 480
Opium, poisoning from, treatment of 142
its external use in croup . 243
eating among the Turks . 294
Mr. Houlton's new preparation of . . . 540
Oppenheim, Dr. on medicine in Turkey 380
Organic structure, unity of . 478
Os uteri, state of, in pregnancy . 455
Ottley, Mr. his Life of John Hunter 75
Ovaria, tumour of, operated on . 477

Palmer, Mr. his edition of Hunter's Works . . . . . 75
Parasites, vegetable . . 18
Parent-Duchatelet on prostitution . 63
Paris, on prostitution in . . ib.
Pectoriloquy, its value . . 322
Percussion, Dr. Stokes on . 283
Pericardium, morbid anatomy of . 41
case of hernia of . 532
Peritoneum, morbid anatomy of . 46
Peritonitis, tubercular . . 49
Perry, Mr. on varicose aneurism . 138
Pharmacopœia of London . . 101
Phillips, Mr. his Pharmacopœia . ib.
Mr. B. on fractures of the atlas . . . 141
Phthisis, Aretæus's account of . 491
Physiological discoveries, by Dr. Todd 281
Plants, on the generation of . 561
Pleura, morbid anatomy of . 43
Pleuritis, chronic, cases of . . 479
Plumbe, Mr. on diseases of the skin 175
Pneumonia of the old . . 501
Dr. Addison on . 356
Dr. Stokes on . . 313
treatment of . . 316
Poisoning by liquor potassæ . 239
by arsenic, Mr. Taylor on 358
its frequency in Turkey . 389
Polypus uteri, remarks on . . 183
Pregnancy, spurious . . 460
extra-uterine, diagnosis of 493
signs and symptoms of . 448
Prolapsus uteri, remarks on . 182
Prostitution in Paris . . 63
Provincial Medical Association 551, 560
Transactions of 477
Puerperal fever, Dr. Michaelis on . 517
Pulse, pulmonic, account of . 241
Dr. Knox on the . . ib.
its estimation by the Turks . 387
Pus in the blood, on detecting . 526

Queen, medical staff of the . 567
Quickening, as a sign of pregnancy 454

PAGE
Quina, mode of disguising the taste of 246

Raspail's Vegetable Physiology . 1
Rectum, on the diseases of . 467
foreign bodies in . 468
Registration of diseases . . 280
Religion, its influence on health . 55
Respiration of plants . . 22
Retina, on the anatomy of . . 499
Retroversion of the uterus . . 410
Reynolds, Dr. his Aretæus . 490
Rheumatism, curious form of . 393
Robertson, Dr. on cataract . 257
Russia, state of medicine in . 263

Sadler, Dr. on moxa . . 217
Sampson, Mr. on tracheotomy in intoxication . . . 140
Sap in vegetables, motion of . 20
Sealy, Dr. his Medical Essays . 196
Secretions of plants . . 26
Sensations, transference of . 443
Scarlatina and smallpox united . 219
its prevention by belladonna 392
Schwann, Dr. on artificial digestion 201
Schwörer, Dr. on infanticide . 87
Shapter, Dr. his case of loss of memory 478
Sherwood, Mr. death of . . 568
Skin diseases, treatises on . . 175
Skull fractured during labour . 233
Societies, scientific, in Norway . 548
Solitary confinement, its effects on health . . . . 191
Solly, Mr. his work on the Brain . 485
Sommer, Dr. on the signs of death 197
Somnambulism, magnetic . . 442
Spender, Mr. on mortification . 259
Spillan, Dr. his Pharmacopœia . 101
Spinal cord, on injuries of . . 143
Dr. Nasse on . . 415
irritation, Dr. Griffin on . 252
Spine, on the state of the muscles in curvature of . . . 509
St. Vitus's dance, Dr. Stiebel on . 505
Stafford, Mr. on dissection wounds . 140
Stethoscope, its importance . 295
Still-born children, statistics of . 234
Stokes, Dr. on disease of the lungs 285
Strains, on the treatment of . 232
Strangulation, intestinal, internal . 168
Suicide, statistics of . . 234
Surgery, Mr. Morgan's principles of 185
state of, in Turkey . 395

PAGE
Syrupus papaveris, adulteration of . 118

Tapeworm, on the cure of . . 112
Taylor, Mr. on arsenical poisoning . 358
Temperature of insects . . 527
Tetanus, case of . . . 478
Tendo Achillis, division of . 231, 539
Thigh-bone, mode of its retention . 205
Thomson, Dr. W. on black expectoration . . . . 150
Throat-cut, singular case of . 515
Thyroid gland, on dropsy of . 478
Tissues, primary, of vegetables . 6
Todd, Dr. his physiological discoveries 281
Tracheotomy in intoxication . 140
Transactions of the Provincial Association . . . . 477
of the Medico-Chirurgical Society . 138
Travers, Mr. B. on dislocation of the thigh . . . . 143
Treviranus, his Vegetable Physiology 1
Tubercles, Dr. Harrison on . . 244
of the lungs, Dr. Stokes on 318
Sebastian and Albers on . 216
Dr. Kingston on . 151
of serous membranes . 53
Tulloch, Mr. on the statistics of negroes 261
Tumour, malignant, case of . 479
Tumours of the mouth, Mr. Liston on 147
Tunica vaginalis, induration of . 172
Turkey, state of medicine in . 380

Ulceration of serous membranes . 51
Umbilicus, state of, in pregnancy . 454
Universities, Bavarian, regulations in 562
Uterus, inversion of . . 412

Vaccination in Norway . . 547
Varicocele, treatment of . . 232
Vascularity, Dr. Yelloly on . 138
Veterinary medicine in Norway . 548
Volition, loss of power of . . 500

Wagner, Dr. on belladonna in ileus . 220
Walker, Mr. his Philosophy of the Eye 496
Ward, Dr. on the "bruit de diable" 245
West Indies, on the fevers of . 160
Wilson, Dr. on spotted fever . 535
Women, on the diseases of . 525

Yelloly, Dr. on vascularity . 138

END OF VOL. IV.

London: Printed by J. and C. Adlard,

Bartholomew Close.

